# Type I interferon responses are impaired in latently HIV infected cells

**DOI:** 10.1186/s12977-016-0302-9

**Published:** 2016-09-09

**Authors:** Nischal Ranganath, Teslin S. Sandstrom, Saleh Fadel, Sandra C. Côté, Jonathan B. Angel

**Affiliations:** 1 Ottawa Hospital Research Institute, ORCC Room C4445, 501 Smyth Road, Ottawa, ON K1H 8L6 Canada; 2 Department of Biochemistry, Microbiology and Immunology, University of Ottawa, Ottawa, ON Canada; 3 Division of Infectious Diseases, Ottawa Hospital-General Campus, Ottawa, ON Canada

**Keywords:** HIV-1, Latency, Interferon stimulated genes (ISG)

## Abstract

**Background:**

The latent HIV-1 reservoir represents the primary barrier to the eradication of HIV-1 infection. The design of novel reservoir-clearance strategies, however, is impeded in part by the inability to distinguish latently HIV-infected cells from uninfected cells. Significant impairment of the type I interferon (IFN-I) response is observed during productive HIV-1 infection. Although this remains poorly described in the context of latent HIV-1 infection, presence of potential defects may serve as a novel therapeutic target. Therefore, IFN-I pathways were characterized using two latently HIV-1-infected cell lines, U1 and OM10.1, in comparison to their respective uninfected parental U937 and HL60 cell lines.

**Findings:**

Constitutive expression and induction of important mediators of IFN-I signaling including IFNα/β cytokines, IFNAR1, MHC-I, ISG15, and PKR were evaluated following exogenous IFNα or poly(I:C) treatment. Differences in basal expression of IFNAR1, MHC-I, and PKR were observed between the latently HIV-1 infected and uninfected cell lines. In parallel, significant impairments in the induction of MHC-I, ISG15 and PKR, as well as secretion of IFNα/β cytokines were observed in response to appropriate exogenous stimulation within the two latently HIV-infected U1 and OM10.1 cells, relative to their HIV-uninfected parental cells.

**Conclusions:**

In comparison to the HIV-uninfected U937 and HL60 cell lines, widespread defects in IFN-I responsiveness were observed within the latently HIV-infected U1 and OM10.1 cells. These impairments represent novel therapeutic targets, which may be amenable to strategies currently employed in cancer therapy.

**Electronic supplementary material:**

The online version of this article (doi:10.1186/s12977-016-0302-9) contains supplementary material, which is available to authorized users.

## Findings

Combination antiretroviral therapy (cART) effectively suppresses viral replication in HIV-1 infected individuals. However, due to the persistence of the virus as integrated proviral DNA in long-lived latent reservoirs, cART fails to eradicate HIV-1 [1, 2]. The latent HIV-1 reservoir not only evades immune surveillance, but can also serve as a source of infectious virus upon treatment cessation. Therefore, identification of potential targets that distinguish latently HIV-1 infected cells from normal cells may be necessary for the development of curative therapies.

Cancer pathogenesis presents a unique platform for studying HIV-1 latency. Anti-proliferative innate immune defenses such as the type I interferon (IFN-I) system exert significant selective pressure on cancers. This often results in tumor cells that have impairments in IFN-I pathways, thereby facilitating their escape from tumor suppressive immune responses [3]. Several defects in IFN-I pathways have been characterized in tumors, including reduction in IFNα/β secretion and IFNα/β-receptor subunit-1 (IFNAR1) expression [4, 5], and altered induction of downstream IFN-stimulated genes (ISGs) such as major histocompatibility complex-I (MHC-I) [6], interferon regulatory factor-3 (IRF3) [7], retinoic acid-inducible gene 1 (RIG-I) [8], and protein kinase R (PKR) [9]. Although such defects promote tumor survival and immune evasion, they can be used to distinguish tumor cells from healthy cells, and have therefore been exploited as therapeutic targets [10].

A significant antiviral IFN-I response is also observed during the acute phase of HIV-1 infection and can effectively suppress viral replication [11]. Sandler and colleagues demonstrated that IFNα-2a treatment prior to SIV infection in rhesus macaques resulted in significant resistance to viral transmission [12]. Interestingly however, the IFN-I response to HIV-1 infection represents a double-edged sword with the capacity to potentiate disease pathogenesis. Within the study, ongoing exposure to IFNα-2a resulted in significant IFN-I desensitization, impaired ISG expression, increased viral load, and accelerated CD4^+^ T cell decline [12]. Similarly, during the chronic phase of HIV-1 infection in vivo, persistent elevation in plasma IFNα has been correlated with higher viral loads and faster disease progression [13]. Therefore, further investigation and elucidation of the complex interplay between HIV-1 and the antiviral IFN-I system is necessary.

Driven by antiviral pressures exerted by the IFN-I system, HIV-1 has evolved countermeasures similar to those seen in cancer cells. For instance, transmitted/founder viruses have been identified to be relatively resistant to inhibition by IFNα, potentially conferring a selective advantage during early infection [14]. In parallel, defects in IFN-I signaling have been demonstrated during productive HIV infection including the inhibition of IRF3 by HIV Vpr [15], disruption of MHC-I by HIV Nef [16], degradation of RIG-I by HIV protease [17], and Tat-mediated impairment of PKR [18]. IFN-I signaling and responsiveness in the context of HIV-1 latency, however, has yet to be characterized. We therefore investigated components of the IFN-I pathway within latently HIV-infected cells, which, if impaired, may facilitate selective eradication using novel treatment approaches currently employed in cancer therapy [10].

Due to the complex mechanisms involved in the establishment of HIV latency [19], none of the existing in vitro models truly recapitulate latency as occurs in vivo. As such, cell line models are often used to delineate features of HIV-1 latency, not only because of the homogenous presence of latently infected cells within the clonal population, but also the capacity to induce viral replication upon appropriate stimulation [20]. Therefore, IFN-I signaling was characterized in the latently HIV-infected U1 [21] and OM10.1 cells [22] (NIH AIDS Reagent Program, Divison of AIDS, NIAID, NIH), as well as the respective parental HIV-uninfected U937 (CRL-1593.2) and HL60 (CCL-240) cells (ATCC, Manassas, VA, USA). Cells were maintained in RPMI-1640 supplemented with 10 % heat-inactivated FBS, penicillin (100 U/mL), and streptomycin (100 μg/mL) at 37 °C and 5 % CO_2_.

Aspects of the IFN-I pathway shown to be altered during productive HIV-1 infection, including IFN-I cytokines (IFNα/β), IFNAR1, and the antiviral ISGs, MHC-I, Interferon stimulated gene-15 (ISG15), and PKR, were investigated. Surface expression of IFNAR1 (Clone-85228, R&D Systems, Minneapolis, MN, USA) and MHC-I (clone-W6/32, eBioscience, San Diego, CA, USA), as well as intracellular expression of ISG15 (clone-851701, R&D Systems) and PKR (clone-6H3A10, Abcam, Toronto, ON, Canada) were quantified by flow cytometry both at basal levels and following stimulation with increasing concentrations of IFNα for 24 h (PBL Assay Science, Piscataway, NJ, USA). In addition, IFNα-induced mRNA expression of ISG15 and PKR was quantified by RT-PCR using the following primers: ISG15 forward (5′-GAGAGGCAGCGAACTCATCT-3′) and reverse (5′-CTTCAGCTCTGACACCGACA-3′) [23] and PKR forward (5′-TCTTCATGTATGTGACACTGC-3′) and reverse (5′-CACACAGTCAAGGTCCTTAG-3′) [24].

### Basal expression of IFNAR1, MHC-I, and PKR is lower in latently HIV-infected cells

**Fig. 1:**
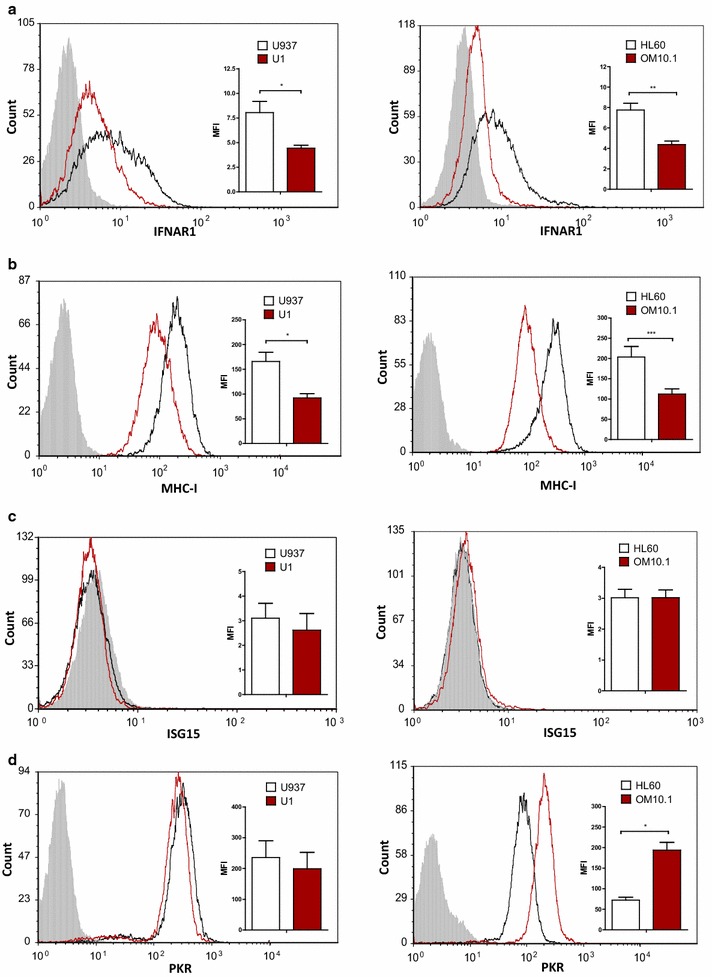
Expression of IFNAR1, MHC-I, ISG15, and PKR in HIV-uninfected and latently HIV-infected cells. Constitutive expression of several ISGs was quantified by flow cytometry in latently HIV-infected U1 and OM10.1 cells. Surface expression of **a** IFNAR1 (n = 5) and **b** MHC-I (n = 6) were significantly lower in the latently HIV-infected U1 and OM10.1 cells, than in uninfected parental controls. **c** Constitutive intracellular expression of ISG15 (n = 6) was minimal in all cell lines. **d** Intracellular PKR expression was higher in latently HIV-infected OM10.1 cells, than in uninfected HL60 cells (n = 6). No difference in basal PKR expression was observed between U937 and U1 cells (n = 6). Representative histogram and cumulative data for each ISG is shown. *p < 0.0001, **p = 0.002, ***p < 0.0017 by unpaired *T* test

The constitutive expression of IFNAR1 was ~twofold lower in the latently HIV-infected U1 and OM10.1 cells, than in the HIV-uninfected controls (Fig. 1a). Additionally, surface expression of MHC-I, an antiviral protein known to be downregulated during HIV replication [16], was demonstrated to be significantly lower in both U1 and OM10.1 cells, than in the respective uninfected parental cells (Fig. 1b). There was minimal constitutive expression of ISG15, an IFN-inducible ubiquitin-like antiviral protein, in all cell lines (Fig. 1c). Similarly, there was no difference in the basal expression of PKR, a double-stranded RNA-sensing pattern recognition receptor (PRR), between U1 and U937 cells. In contrast, OM10.1 cells had higher expression of PKR in comparison to the HIV-uninfected HL60 cells (Fig. 1d). This pattern of PKR expression for both cell lines pairs was confirmed by Western blot (Additional file 1). In summary, differences in basal levels of IFNAR1, MHC-I, and PKR, were observed between two independent latently HIV-infected and uninfected cell line pairs.

### Responsiveness to exogenous IFNα is impaired in latently HIV-infected cells

**Fig. 2:**
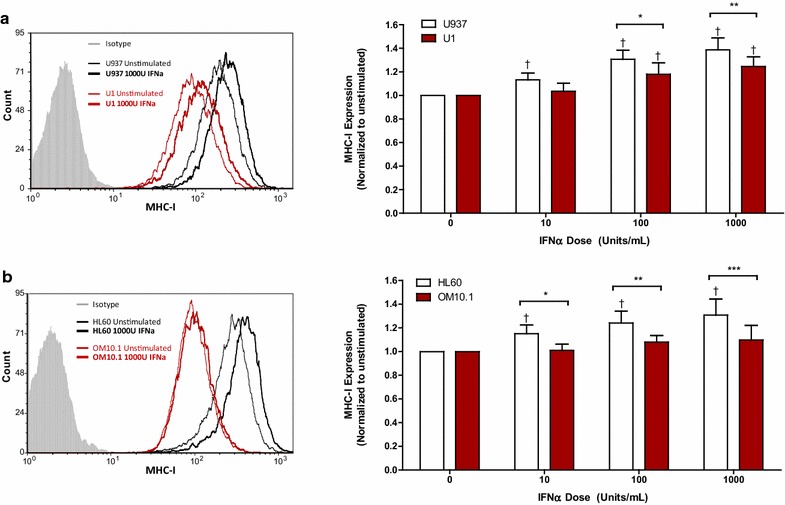
IFNα-induced expression of MHC-I is impaired in latently HIV-infected U1 and OM10.1 cells. Cell lines were stimulated with 10, 100, or 1000 U/mL of exogenous IFNα for 24 h. Following stimulation, cells were collected and surface expression of MHC-I was assessed by flow cytometry. Representative histogram and summary data of IFNα-induced MHC-I expression normalized to unstimulated controls is shown for **a** U937 and U1 cells (n = 6) and **b** HL60 and OM10.1 cells (n = 6). ^†^ *p* < 0.0001 by one-way ANOVA and *p* < 0.05 by pairwise Dunnett’s test compared to unstimulated cells. *p < 0.05; **p < 0.01, and ***p < 0.001 by Two-way ANOVA with Bonferroni post-test for multiple comparisons

**Fig. 3:**
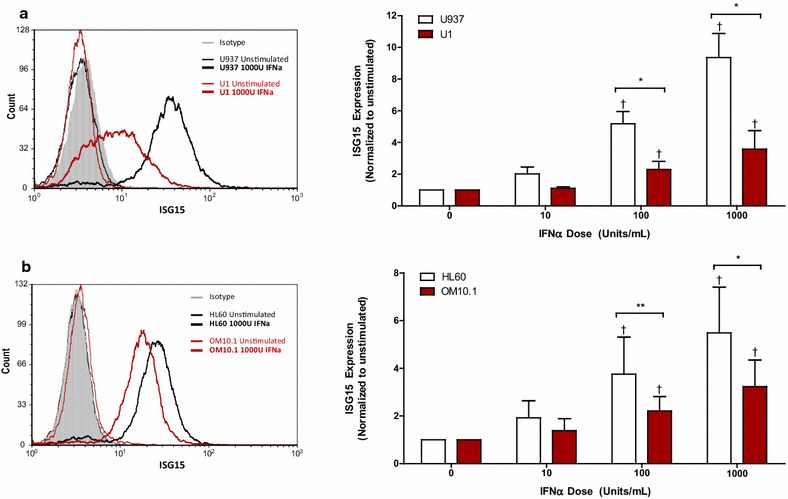
IFNα-induced expression of ISG15 is impaired in latently HIV-infected U1 and OM10.1 cells. Cell lines were treated with increasing concentrations of exogenous IFNα for 24 h. Following stimulation, cells were fixed and permeabilized, after which intracellular ISG15 expression was measured by flow cytometry. Representative histogram and cumulative data of IFNα-induced ISG15 expression normalized to unstimulated controls is shown for **a** U937 and U1 cells (n = 6) and **b** HL60 and OM10.1 cells (n = 8). ^†^ *p* < 0.0001 by one-way ANOVA and *p* < 0.05 by pairwise Dunnett’s Test compared to unstimulated cells. *p < 0.001; **p < 0.05 by Two-way ANOVA with Bonferroni post-test for multiple comparisons

**Fig. 4:**
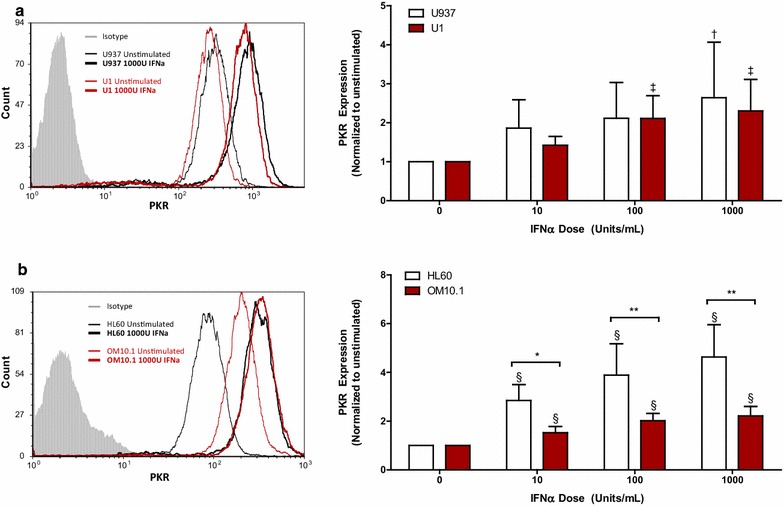
IFNα-induced PKR expression was impaired in latently HIV-infected OM10.1 cells. Cell lines were left unstimulated or treated with increasing concentrations of exogenous IFNα for 24 h. Following stimulation, cells were fixed and permeabilized, after which intracellular PKR expression was measured by flow cytometry. Representative histogram and cumulative summary of IFNα-induced PKR expression, normalized to unstimulated controls, is shown for **a** U937 and U1 cells (n = 6) and **b** HL60 and OM10.1 cells (n = 6). ^†^ *p* = 0.04; ^‡^ *p* = 0.0008; ^§^ *p* < 0.0001 by one-way ANOVA and *p* < 0.05 by pairwise Dunnett’s Test compared to unstimulated cells. *p < 0.05; **p < 0.001 by Two-way ANOVA with Bonferroni post-test for multiple comparisons

Next, the responsiveness of latently HIV-infected cells to exogenous IFNα was investigated by characterizing the expression of downstream ISGs. MHC-I expression was upregulated in response to IFNα in all cell lines, but was significantly lower in the latently HIV-infected U1 (Fig. 2a) and OM10.1 cells (Fig. 2b) when compared to their respective controls. Similarly, IFNα enhanced the expression of ISG15 in a dose-dependent manner in all cell lines, but the level of ISG15 expression was lower in the latently HIV-infected U1 (Fig. 3a) and OM10.1 cells (Fig. 3b) than in the HIV-uninfected controls. Although no difference in the expression of ISG15 mRNA was observed between U937 and U1 cells following IFNα treatment, a significantly lower level of ISG15 expression was observed in OM10.1 cells relative to HL60 cells (Additional file 2A). Finally, IFNα-induced PKR expression was found to be impaired in OM10.1 cells relative to HL60 cells (Fig. 4b), but did not differ between U1 and U937 cells (Fig. 4a). Consistent with this, lower levels of IFNα-induced PKR gene expression were observed in OM10.1 cells than in HL60 cells, but no difference was observed between U1 and U937 cells (Additional file 2B).

### Poly(I:C)-induced activation of IFN-I pathways is defective in latently HIV-infected cells

The observed impairments in IFNα-induced ISG expression in latently HIV-infected U1 and OM10.1 cells compared to healthy parental controls can potentially be explained by differences in IFNAR1 expression (Fig. 1a). To address this possibility, cell lines were transfected with the synthetic RNA analog, polyinosinic: polycytidylic acid (poly(I:C)). Recognition of poly(I:C) by various intracellular RNA-sensing PRRs, including toll-like receptor-3, PKR, RIG-I, and melanocyte-differentiation factor 5, has been shown to directly induce ISG expression [25]. Therefore, transfection of cells with poly(I:C) provided a means by which to measure ISG induction in the absence of exogenous IFN-I stimulation.

**Fig. 5:**
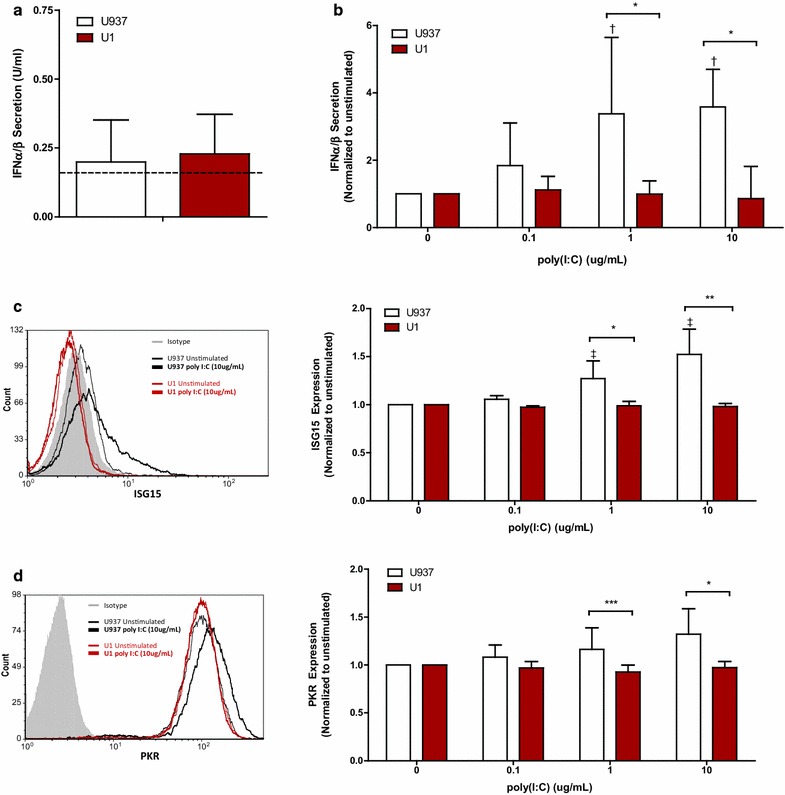
Responsiveness to poly(I:C) was defective in U1 cells, when compared to U937 cells. U937 and U1 cells were transfected (Lipofectamine^®^2000) with media alone or 0.1, 1, or 10ug/mL of poly(I:C) for 48 h. **a** Minimal constitutive secretion of of IFNα/β (n = 5) was observed in U937 and U1 cells, as quantified using using the HEK-Blue™ IFNα/β biologic assay (dashed line denotes lower limit of detection of assay = 0.15 U/mL). **b** Secretion of IFNα/β by U937 cells, but not latently HIV-infected U1 cells was detected following poly(I:C) transfection (n = 5). **c** Poly(I:C) induced ISG15 expression (n = 5), as measured by flow cytometry, was observed only in the HIV-uninfected U937 cells. **d** Similarly, poly(I:C) induced expression of PKR (n = 5) was impaired in U1 cells, but not in U937 cells. Representative histogram and cumulative summary of poly(I:C)-induced ISG expression is shown. ^†^ *p* = 0.03; ^‡^ *p* = 0.0004 as measured by one-way ANOVA and *p* < 0.05 by pairwise Dunnett’s Test compared to unstimulated cells. *p < 0.01; **p < 0.001, ***p < 0.05 by Two-way ANOVA with Bonferroni post-test for multiple comparisons

Using the Lipofectamine^®^2000 reagent (ThermoFisher Scientific, Waltham, MA, USA), cells were transfected with various concentrations of poly (I:C) (InvivoGen, San Diego, CA, USA) for 48 h. Similar levels of transfection efficiency were confirmed in all cell lines using Rhodamine labelled poly(I:C) (Invivogen). Following stimulation, IFNα/β secretion was assessed using the HEK-Blue™ IFNα/β (InvivoGen) biologic assay as described [26], and intracellular ISG15 and PKR expression were assessed as before. Low levels of endogenous IFNα/β were detectable in culture supernatant of unstimulated U937 and U1 cells (Fig. 5a). However, upregulation of IFNα/β production in response to poly(I:C) transfection was only observed in U937 cells (Fig. 5b). While poly(I:C) caused a dose-dependent increase in intracellular ISG15 expression in U937 cells, no effect was observed in the latently HIV-infected U1 cells (Fig. 5c). Similarly, poly(I:C)-induced PKR expression was impaired in the U1 cells, when compared to U937 cells (Fig. 5d). Similar defects in IFN-I pathway induction following poly(I:C) stimulation were also quantified within HL60 and OM10.1 cells (Additional file 3).

Utilizing two independent cell line models of HIV-latency, we have demonstrated widespread IFN-I pathway defects, including impaired secretion of IFNα/β and expression of IFNAR1, MHC-I, ISG15, and PKR, following exogenous IFNα or poly(I:C) stimulation. Interestingly, antagonism of IFNAR1 in rhesus macaques in the context of SIV infection resulted in significant impairment in ISG expression, particularly in the pathways associated with PRRs [12]. The observed defects in IFNAR1 in latently HIV-1 infected cells may therefore not only promote escape from IFN-mediated immune responses, as reported in several tumors [5], but also facilitate the establishment of the latent reservoir. ISG15 plays critical antiviral roles both by regulating IFN-I mediators such as RIG-I and IRF3, as well as through direct inhibition of viral proteins [27]. Impaired expression of ISG15 in latently HIV-infected U1 and OM10.1 cells may therefore contribute to downstream defects in the activation of IFN-I pathways crucial for antiviral defense. In addition, impaired induction of MHC-I and PKR can contribute to abnormalities in viral sensing and antigen presentation, which may facilitate the establishment and maintenance of HIV latency. Interestingly, we observed an impaired induction of PKR in OM10.1 cells, but not in U1 cells. The higher constitutive expression of PKR in OM10.1 cells when compared to HL60 cells may in part contribute to the impaired induction observed. Given the importance of HIV-1 Tat in inhibiting PKR activation, similar PKR induction observed in U937 and U1 cells may be explained by the presence of defects in Tat in U1 cells [18, 28].

The underlying mechanism(s) for the defective IFN-I response observed within the latently HIV-infected cell line models employed have yet to be elucidated. Similar defects in IFN-I signaling have previously been observed during productive HIV-1 infection, largely mediated by viral proteins including Tat, Vpu, Vif, and Nef. Although latent HIV-1 infection in U1 and OM10.1 cells is characterized by minimal p24 antigen expression (Additional file 4), low-level gene transcription and expression of viral proteins, including those known to interfere with IFN-I signaling, may be present [22, 29]. A recent report demonstrated that a Tat-inhibitor can further suppress OM10.1 cells into a state of ‘deep latency’ marked by transcriptional silence, thereby suggesting that within models of HIV latency viral proteins such as Tat may be present and functional [30]. Consistent with this, Pace and colleagues have demonstrated the presence of viral transcripts encoding Gag, Env, Vif, and Tat/Rev within their primary CD4^+^ T cell model of HIV latency [31]. An alternate explanation for our observations may be that the initial HIV-1 infection induces permanent changes to the cell, resulting in IFN-I defects that persist during latency.

Further studies investigating defects in the IFN-I pathway in the setting of HIV latency, as well as elucidating the underlying mechanisms for such alterations will be necessary. Nevertheless, the defects in IFN-I signaling and responsiveness reported here may serve as novel therapeutic targets in the search for HIV-1 eradication strategies.

## Additional files

10.1186/s12977-016-0302-9 Constitutive PKR expression was higher in OM10.1 cells than in HL60 cells, but similar between U937 and U1 cells. Basal expression of PKR was assessed in **A.** U937 and U1 cells (n = 5) and **B.** HL60 and OM10.1 cells (n = 4) by Western Blot using primary (sc-6282, Santa Cruz Biotechnology, Dallas, TX, USA) and secondary antibodies (HAF007, R&D Systems, Minneapolis, MN, USA). β-actin was used as the loading control. Representative blots are shown.
10.1186/s12977-016-0302-9 IFNα-induced ISG15 and PKR mRNA expression was lower in OM10.1 cells than HL60 cells, but no difference was observed between U1 and U937 cells. Cell lines were left unstimulated or treated with increasing concentrations of exogenous IFNα for 16 h. Cell-associated *ISG15* and *PKR* mRNA expression was then quantified by RT-PCR. *GAPDH* and *RPS18* (Prime PCR, BioRad) were used as reference genes. **A.** mRNA expression of ISG15 normalized to unstimulated controls is shown for U937/U1 (n = 4) and HL60/OM10.1 (n = 4) cell lines pairs. **B.** mRNA expression of PKR normalized to unstimulated controls is shown for U937/U1 (n = 4) and HL60/OM10.1 (n = 4) cell lines pairs. † p < 0.0001, ‡ p = 0.0015, § p = 0.043 by one-way ANOVA and p < 0.05 by pairwise Dunnett’s Test compared to unstimulated cells. *p < 0.05 and **p < 0.0001 by two-way ANOVA with Bonferroni post-test for multiple comparisons.
10.1186/s12977-016-0302-9 Poly(I:C)-induced expression of ISG15 and PKR was impaired in OM10.1 cells, but not in HL60 cells. HL60 and OM10.1 cells were transfected with increasing doses of poly(I:C) for 48 h as previously described. **A.** Both induction and level of expression of ISG15 (n = 5) was significantly lower in the OM10.1 cells when compared to HIV-uninfected HL60 cells. **B.** Although not significant, the qualitative PKR expression was lower in the latently HIV-1 infected OM10.1 cells than in HL60 cells (n = 6) in response to poly(I:C). *p < 0.05 by two-way ANOVA with Bonferroni post-test for multiple comparisons.
10.1186/s12977-016-0302-9 Minimal constitutive p24 antigen expression was observed in latently HIV-infected U1 and OM10.1 cells. Intracellular expression of HIV-1 p24 antigen (6604667, Beckman Coulter, Mississauga, Ontario, Canada) was quantified by flow cytometry. Minimal basal expression of p24 antigen was detected in the latently HIV-1 infected **A.** U1 (n = 7) and **B.** OM10.1 cells (n = 6). Representative dot plot and gating strategy, as well as summative data of intracellular p24 expression in both latently infected cell lines is shown.

## Abbreviations

cART: Combination antiretroviral therapy
IFN-I: Type I interferon
IFNAR1: IFNα/β-receptor subunit-1
ISG: Interferon stimulated gene
MHC-I: Major histocompatibility complex-I
IRF3: Interferon regulatory factor-3
RIG-I: Retinoic acid-inducible gene 1
PKR: Protein kinase R
ISG15: Interferon stimulated gene-15
PRR: Pattern recognition receptor
Poly(I:C): Polyinosinic: polycytidylic acid
