# Supplementary figures and images for: Type I interferon responses are impaired in latently HIV infected cells
Source: Retrovirology. 2016 Sep 9;13(1):66. doi: 10.1186/s12977-016-0302-9 (PMC5017046; doi:10.1186/s12977-016-0302-9)

## Slide 1
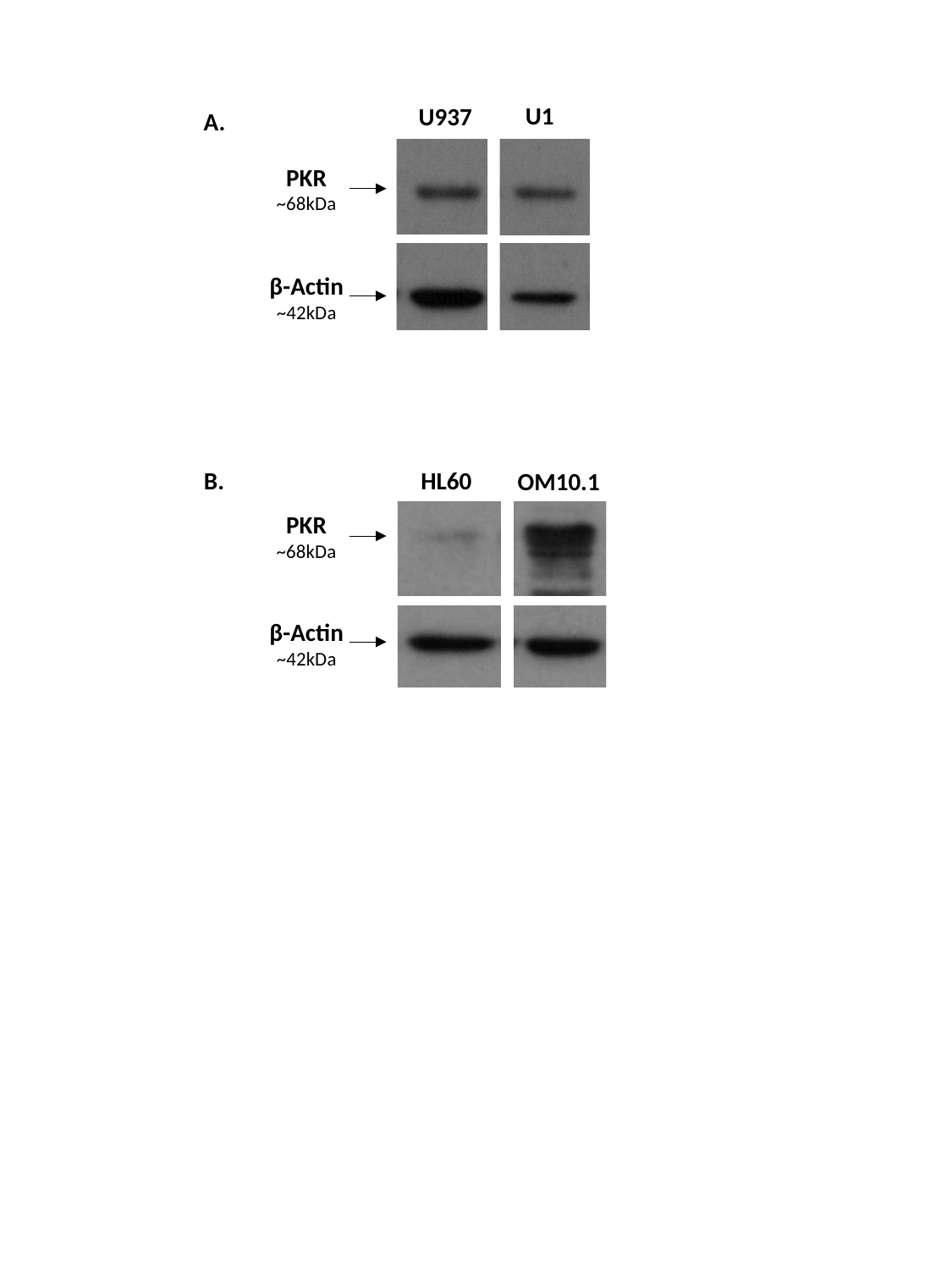

U1
U937
A.
PKR
~68kDa
β-Actin
~42kDa
B.
HL60
OM10.1
PKR
~68kDa
β-Actin
~42kDa

Supplement: Supplementary file 1 — 10.1186/s12977-016-0302-9 Constitutive PKR expression was higher in OM10.1 cells than in HL60 cells, but similar between U937 and U1 cells. Basal expression of PKR was assessed in A. U937 and U1 cells (n = 5) and B. HL60 and OM10.1 cells (n = 4) by Western Blot using primary (sc-6282, Santa Cruz Biotechnology, Dallas, TX, USA) and secondary antibodies (HAF007, R&D Systems, Minneapolis, MN, USA). β-actin was used as the loading control. Representative blots are shown. [file 12977_2016_302_MOESM1_ESM.pptx]

## Slide 1
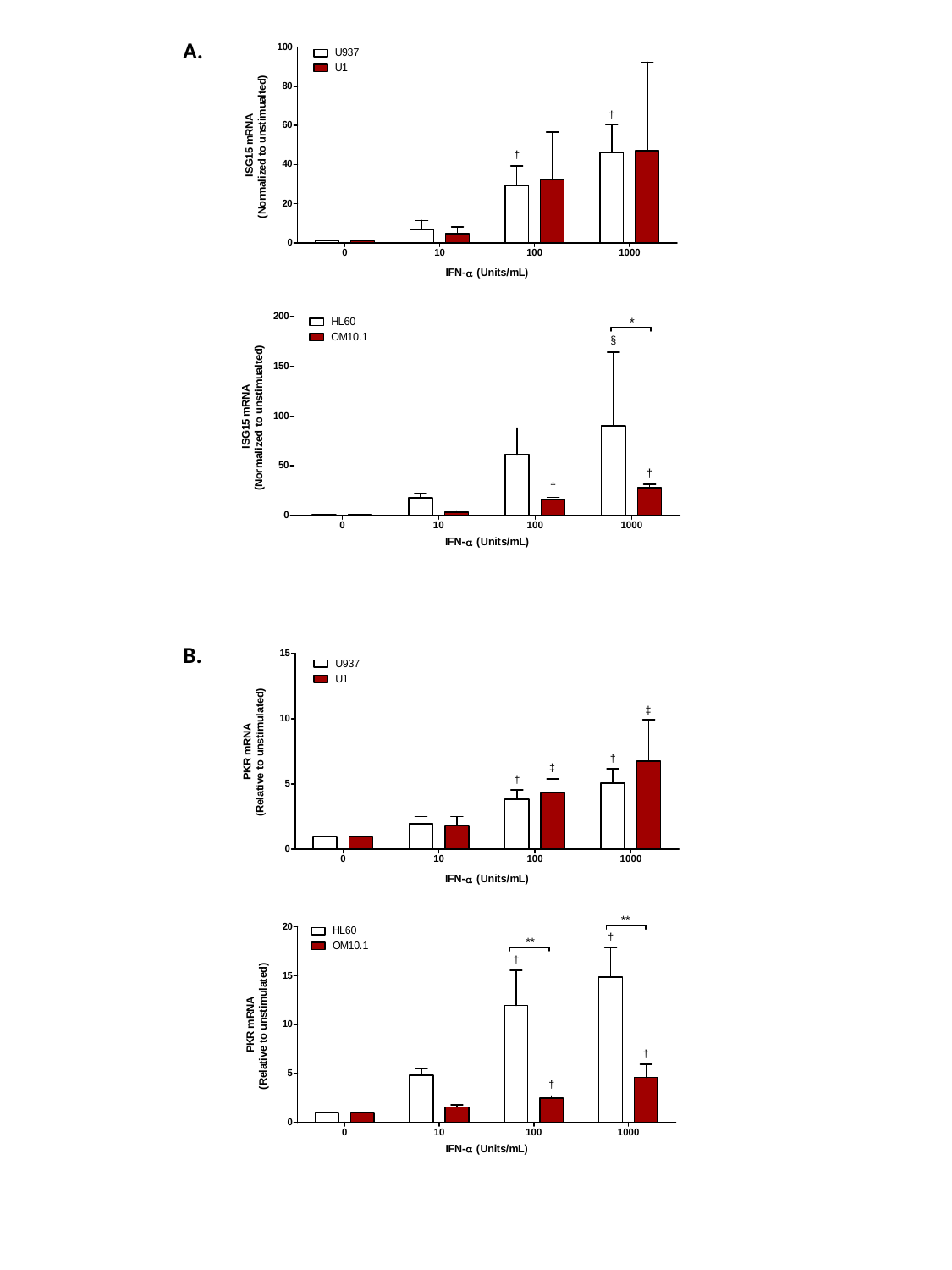

A.
B.

Supplement: Supplementary file 2 — 10.1186/s12977-016-0302-9 IFNα-induced ISG15 and PKR mRNA expression was lower in OM10.1 cells than HL60 cells, but no difference was observed between U1 and U937 cells. Cell lines were left unstimulated or treated with increasing concentrations of exogenous IFNα for 16 h. Cell-associated ISG15 and PKR mRNA expression was then quantified by RT-PCR. GAPDH and RPS18 (Prime PCR, BioRad) were used as reference genes. A. mRNA expression of ISG15 normalized to unstimulated controls is shown for U937/U1 (n = 4) and HL60/OM10.1 (n = 4) cell lines pairs. B. mRNA expression of PKR normalized to unstimulated controls is shown for U937/U1 (n = 4) and HL60/OM10.1 (n = 4) cell lines pairs. † p < 0.0001, ‡ p = 0.0015, § p = 0.043 by one-way ANOVA and p < 0.05 by pairwise Dunnett’s Test compared to unstimulated cells. *p < 0.05 and **p < 0.0001 by two-way ANOVA with Bonferroni post-test for multiple comparisons. [file 12977_2016_302_MOESM2_ESM.pptx]

## Slide 1
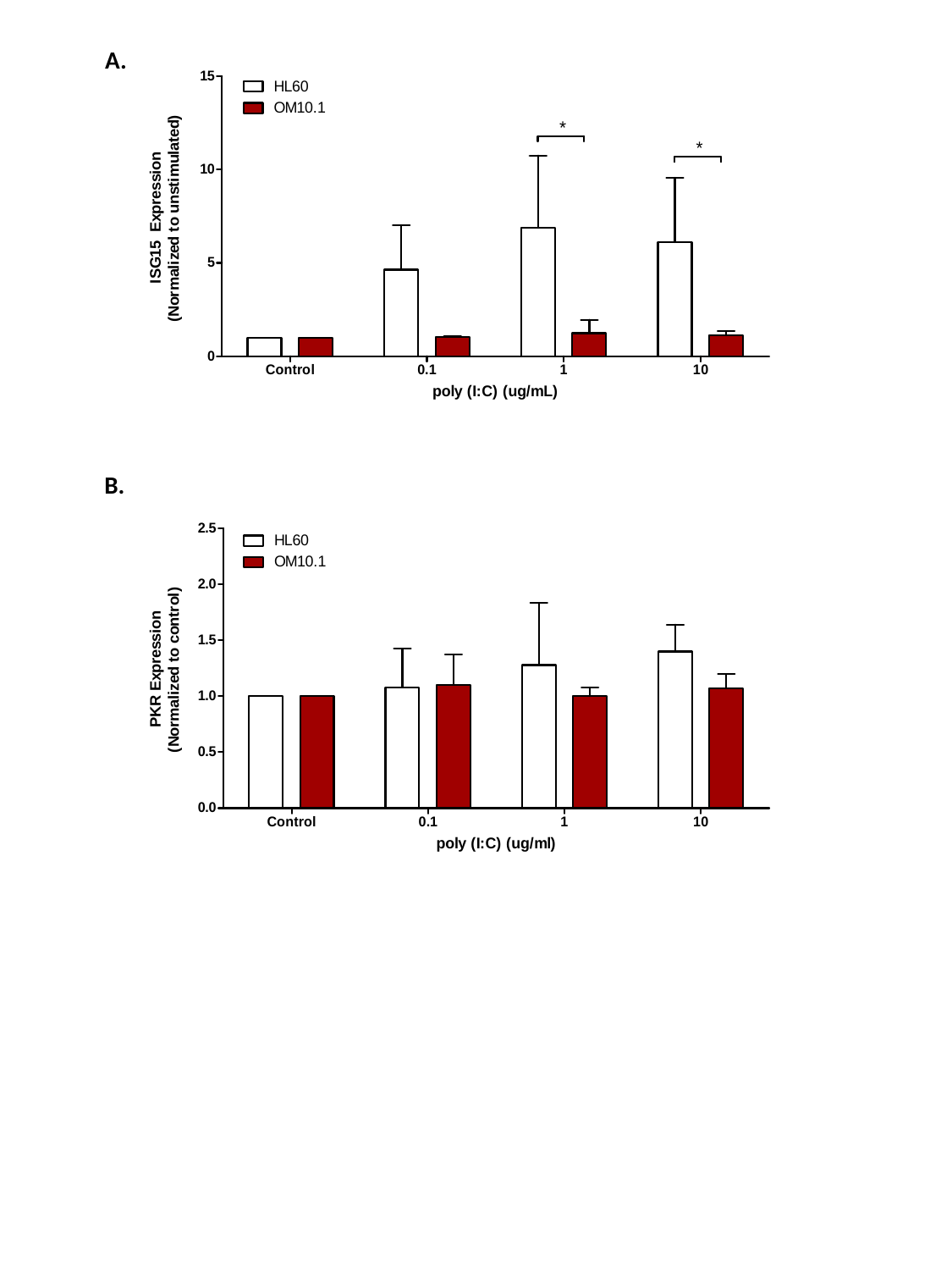

A.
B.

Supplement: Supplementary file 3 — 10.1186/s12977-016-0302-9 Poly(I:C)-induced expression of ISG15 and PKR was impaired in OM10.1 cells, but not in HL60 cells. HL60 and OM10.1 cells were transfected with increasing doses of poly(I:C) for 48 h as previously described. A. Both induction and level of expression of ISG15 (n = 5) was significantly lower in the OM10.1 cells when compared to HIV-uninfected HL60 cells. B. Although not significant, the qualitative PKR expression was lower in the latently HIV-1 infected OM10.1 cells than in HL60 cells (n = 6) in response to poly(I:C). *p < 0.05 by two-way ANOVA with Bonferroni post-test for multiple comparisons. [file 12977_2016_302_MOESM3_ESM.pptx]

## Slide 1
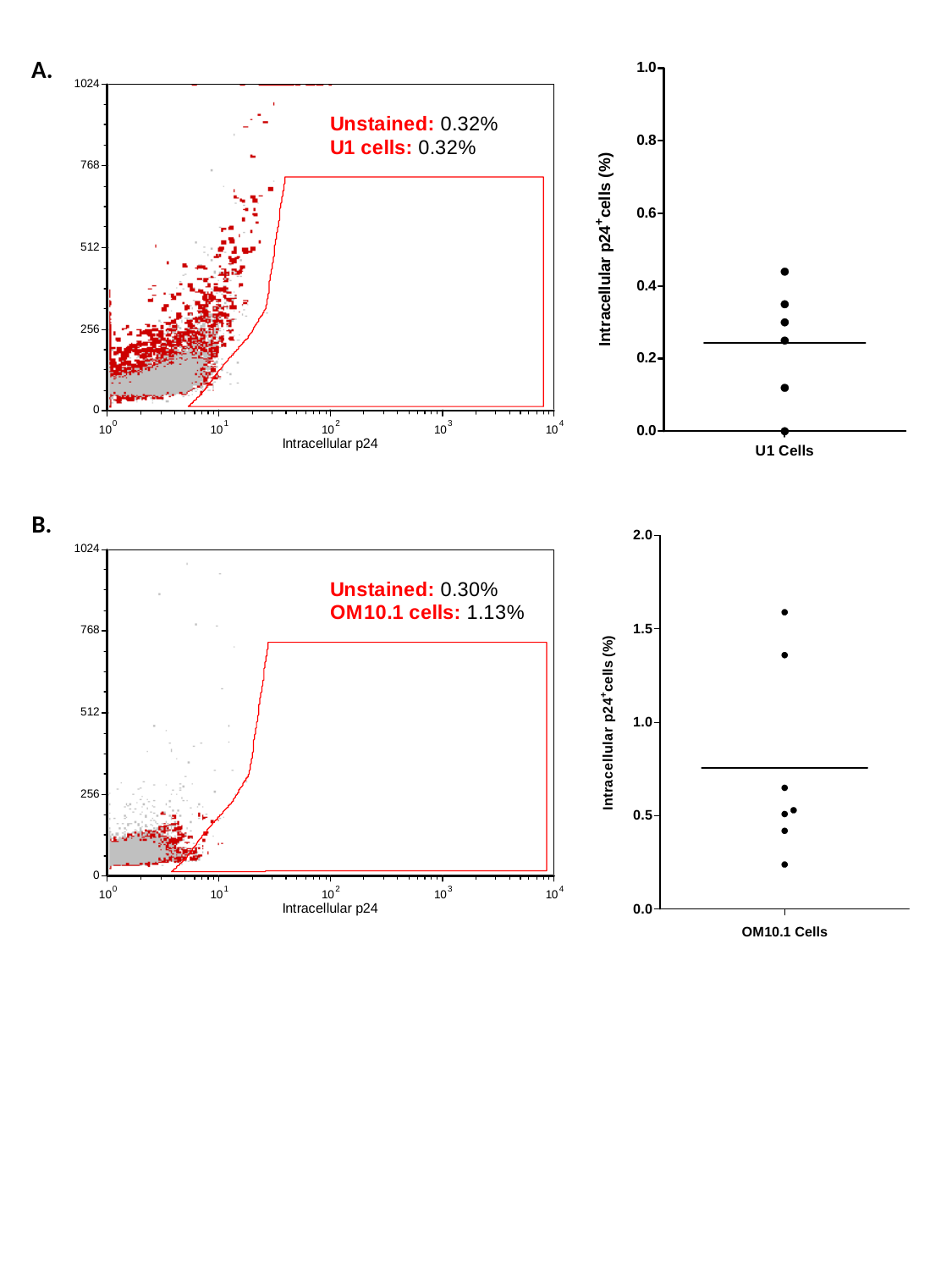

A.
B.

Supplement: Supplementary file 4 — 10.1186/s12977-016-0302-9 Minimal constitutive p24 antigen expression was observed in latently HIV-infected U1 and OM10.1 cells. Intracellular expression of HIV-1 p24 antigen (6604667, Beckman Coulter, Mississauga, Ontario, Canada) was quantified by flow cytometry. Minimal basal expression of p24 antigen was detected in the latently HIV-1 infected A. U1 (n = 7) and B. OM10.1 cells (n = 6). Representative dot plot and gating strategy, as well as summative data of intracellular p24 expression in both latently infected cell lines is shown. [file 12977_2016_302_MOESM4_ESM.pptx]
